# Public awareness, specific knowledge, and worry about mpox (monkeypox): A preliminary community-based study in Shenzhen, China

**DOI:** 10.3389/fpubh.2023.1077564

**Published:** 2023-02-14

**Authors:** Fangmei Ren, Junchao Liu, Jianping Miao, Yucheng Xu, Ruiyin Zhang, Jingjie Fan, Wei Lin

**Affiliations:** ^1^Gushu Community Health Service Center, Baoan Central Hospital of Shenzhen, Shenzhen, China; ^2^Haicheng Community Health Service Center, Baoan Central Hospital of Shenzhen, Shenzhen, China; ^3^Emergency Office, Futian District Center for Disease Control and Prevention, Shenzhen, China; ^4^Department of Programme Immunization, Futian District Center for Disease Control and Prevention, Shenzhen, China; ^5^Department of Preventive Healthcare, Affiliated Shenzhen Maternity and Child Healthcare Hospital, Southern Medical University, Shenzhen, China; ^6^Department of Healthcare, Affiliated Shenzhen Maternity and Child Healthcare Hospital, Southern Medical University, Shenzhen, China

**Keywords:** awareness, knowledge, worry, community resident, mpox (monkeypox)

## Abstract

**Background:**

The mpox (monkeypox) outbreak has been declared to be a public health emergency of international concern by the Director-General of World Health Organization in July 2022. However, evidence regarding the awareness, knowledge, and worry about mpox in the general population remains scant.

**Methods:**

A community-based survey targeting community residents was preliminarily conducted in Shenzhen, China in August 2022 by using a convenience sampling method. Information on mpox-related awareness, knowledge, and worry was collected from each participant. Binary logistic regression analyses with the stepwise procedure were applied to explore the factors associated with awareness, knowledge, and worry about mpox.

**Results:**

A total of 1028 community residents were included in the analysis (mean age: 34.70 years). Among these participants, 77.9% had ever heard of mpox, and 65.3% were aware of the global outbreak of mpox. However, only about half of them had a high level of knowledge regarding mpox (56.5%) and related symptoms (49.7%). More than one-third of them (37.1%) expressed a high level of worry about mpox. Having high knowledge levels of mpox and related symptoms were positively associated with a high level of worry (OR: 1.79, 95%CI: 1.22~2.63 for a single high knowledge level; OR: 1.98, 95%CI: 1.47~2.66 for both high knowledge levels).

**Conclusions:**

This study identified the gaps in public awareness and specific knowledge of mpox in Chinese people, providing scientific evidence for the prevention and control network of mpox at the community level. Targeted health education programs are of urgent need, which should be implemented along with psychological interventions to release public worry if necessary.

## 1. Introduction

The world was greatly impacted by the pandemics caused by infectious diseases in recent years. In 2022, a neglected disease called monkeypox rages and poses a threat to human health, which has been recommended to be replaced with the term of “mpox” by World Health Organization (WHO) (1). Mpox is known to be a zoonotic and infectious disease caused by the mpox virus. This virus is closely related to the smallpox virus since both of them belong to the genus orthopoxvirus (2). This disease is usually endemic in West or Central Africa in recent decades (3). However, many cases have been reported from countries in Europe and North America where the disease was not endemic since early May 2022. The Director-General of WHO declared on July 23 2022 that the multi-country outbreak of mpox was a public health emergency of international concern (4). As of August 18 2022, a total of 39,110 confirmed cases (including 12 deaths) have been reported to WHO from 94 member states (5). The current outbreak of mpox has drawn public attention to this neglected disease as the population is generally susceptible to this disease. It has been revealed that mpox spreads from person to person through direct contact with lesions on the skin or indirect contact with contaminated fomites (2). According to recent reports, most incident cases were men who have sex with men (MSM) that have had recent sexual or close intimate contact, which resulted in an ongoing unprecedented community transmission (6–8). Therefore, raising public awareness about mpox and providing useful recommendations on how to limit further spread among people are of great urgency.

Public awareness and knowledge are crucial for disease prevention and control in the community. Lessons learned from the fight against the Coronavirus disease 2019 (COVID-19) pandemic have suggested a positive role of disease knowledge in facilitating preventive practices (9). Raising the acceptance of preventive measures, including vaccination, could help to prevent infection and the development of disease (10, 11). As a neglected disease, mpox should have the priority to be widely aware and understood by the general population, which helps to promote public cooperation and strengthen society-wide efforts in disease prevention and control. In this context, assessment of awareness and knowledge is so essential for the experts to guide the public to capture useful and correct information in an unbiased manner. Only a few previous studies have assessed the knowledge of mpox among physicians, health school, or university students, in which unsatisfactory knowledge levels and gaps were detected (12–15). Moreover, evidence regarding the awareness and knowledge of mpox in the general population remains scant. Investigations based on population-based studies are urgently needed.

It is noteworthy that the current outbreak of mpox may cause a general state of worry. Worrying about the occurrence of a disease pandemic is a key factor of public health significance. On the one hand, worry helps to promote protective health behaviors and contributes to the effective prevention and control of infectious diseases (16, 17). On the other hand, worry affects people's mental health as researchers have found during the COVID-19 pandemic (18, 19), which may be originated from stringent control measures (e.g., strict home quarantine, prohibition of gathering) and subsequently adverse impacts on the economy and people's daily routines (20). Previously researchers have detected a low level of worry about mpox in Italian adults (21), however, studies reporting mpox related worry in other countries are still lacking. Given the global outbreak and frequent population flows across countries, it's necessary to explore the public worry in the general population.

Based on a community-based survey in Shenzhen city, this study aimed to investigate the awareness, specific knowledge, and worry about mpox, as well as to find out potential associated factors among Chinese people. These investigations will be helpful to deliver coping strategies to reinforce the prevention and control network of mpox at the community level.

## 2. Methods

### 2.1. Study design

This is an analytical cross-sectional study followed by the Strengthening the Reporting of Observational Studies in Epidemiology (STORBE) guidelines (22).

### 2.2. Study settings

In August 2022, a cross-sectional survey using a convenience sampling method has been conducted in the Gushu and Haicheng community health service centers of the Baoan district in Shenzhen, China. During the survey period, attenders in these two community health service centers were presented with a survey recruitment notice. The notice marked the study backgrounds, objectives, potential risks and benefits, and question contents formally. A two-dimensional code linked to an online questionnaire was attached at the bottom of the recruitment notice. People who were interested in this survey could access the questionnaire by scanning the two-dimensional code with their smartphones.

### 2.3. Participants

The inclusion criteria of study participants were: visiting above-mentioned survey sites, age from 18 to 60 years, living in Shenzhen city, and voluntary participation. People who could not read or understand the electronic questionnaire were excluded. A confirmation of voluntary participation for informed consent was required before entering the answer interface. The sample quantity was calculated using the formula of the cross-sectional study: *n* = $\mu_{\text{α}}^{2}$p(1–p)/δ^2^. Here, α = 0.05 (two sides), μ_α_ = 1.96, δ = 0.03, and the awareness rate of mpox *p* = 73.33% (according to the pilot study). The required sample size was 836. With the no-response rate controlled within 10%, the final sample size was determined to be 920. Between August 5 and August 15, 2022, 1054 community residents clicked the survey link, and 1028 of them met the inclusion criteria and completed the survey.

### 2.4. Instrument

An online self-administered questionnaire was applied to collect data. The questionnaire was prepared in Chinese and designed with a variety of information, including individual characteristics on socio-demography, health conditions, daily health habits, awareness/knowledge/worry about mpox, etc. It has been reviewed and corrected by six professionals in public health (two major in community health, three major in infectious diseases control, and one major in epidemiology and psychological healthcare). A pilot study has been performed among 35 community residents to identify the intelligibility of this questionnaire. All participants in the pilot study confirmed that the content of the questionnaire was clear to read and easy to understand.

### 2.5. Measures

#### 2.5.1. Demographic characteristics, health conditions, and daily habits

In the self-administrated questionnaire, participants were required to provide information about demographic characteristics, health conditions, and daily habits. Demographic characteristics included age, gender, household registration, marital status, educated level, employment status, monthly income level, and health insurance. Participants were also asked to recall the history of chronic diseases (e.g., hypertension, diabetes, hyperlipidemia) and overweight/obesity diagnosed by a physician. In addition, health habits in daily routines were collected, including smoking, drinking, physical exercise, and family doctor contracted status.

#### 2.5.2. Awareness of mpox and the global outbreak

As WHO had not yet changed the name of “monkeypox” to “mpox” during the survey time of this study, the term of “monkeypox” was applied in the survey questions. The awareness of mpox was evaluated by asking “*Before the survey time, have you ever heard of monkeypox? (yes/no)*.” People who selected “yes” were regarded to be aware of mpox and were further asked to recall where to learn about mpox. Participants were also required to answer “*Before the survey time, have you ever heard that WHO has declared the global monkeypox outbreak representing a public health emergency of international concern on July 23 2022? (yes/no)*.” Similarly, people who answered “yes” were regarded to be aware of the global outbreak of mpox.

#### 2.5.3. Specific knowledge of mpox and related symptoms

People who were aware of mpox were further assessed for their specific knowledge. The common knowledge of mpox was measured by six knowledge items, including “*A1: Monkeypox is an infectious disease*,” “*A2: Monkeypox is caused by a virus*,” “*A3: People can get monkeypox by close contact with an infected person or animal*,” “*A4: People are generally susceptible to monkeypox*,” “*A5: There are currently no specific treatments for monkeypox*,” and “*A6: There is a vaccine that protects against monkeypox*.” Other six question items were applied to measure the awareness of the clinical symptoms in infected individuals, including “*B1: Monkeypox can cause fever,” “B2: Monkeypox can cause headache*,” “*B3: Monkeypox can cause fatigue or exhaustion*,” “*B4: Monkeypox can cause swollen lymph nodes*,” “*B5: Monkeypox can cause a body rash*,” and “*B6: Monkeypox can cause back and muscle aches*.” These knowledge items were all correct statements, which were developed and adapted from authoritative health education information (23, 24). For each item, participants were required to select one of three options (yes/no/don't know). The answer “don't know” was considered an incorrect answer. The number of right answers ≥ 4 out of six items (correct rate ≥ 66.7%) was regarded as a high level of specific knowledge, whereas people who were not aware of mpox were regarded with a low knowledge level. The construct validity and reliability of two six-item knowledge scales were tested in the pilot study (*n* = 35). Both scales showed satisfactory Kaiser-Meyer-Olkin (KMO) and Bartlett's test of sphericity values (A1–A6: 0.904, χ^2^ = 370.05, *P* < 0.001; B1–B6: 0.905, χ^2^ = 377.924, *P* < 0.001). Two six-item scales were extracted with 93.465 and 93.376% of the total variance in exploratory factor analyses, respectively. The internal consistencies of knowledge items for mpox and related symptoms were also satisfactory in the pilot group (Cronbach's α: 0.984 and 0.986, respectively).

#### 2.5.4. Worry about mpox

In this study, worry about mpox was measured by a single question “*At this moment, how worried are you that monkeypox may cause a global pandemic like COVID-19?*”. In line with previous studies on COVID-19 (18, 25, 26), a five-point response was provided to assess the level of worry, which was described in a progressive manner (1 = not at all worried, 2 = not so worried, 3 = somewhat worried, 4 = very worried, and 5 = extremely worried). Here, people who selected very or extremely worried were regarded with a high level of worry about mpox.

### 2.6. Ethics

The study protocol was approved by the Institutional Review Board of Baoan Central Hospital of Shenzhen and in accordance with the ethical standards of the Declaration of Helsinki. Informed consent was obtained from all participants for the study.

### 2.7. Statistical analyses

As all questions were set up to be fully answered before submission, there was no missing data in the final dataset. Descriptive statistical analyses were conducted, showing counts and frequencies for categorical variables. Chi-square tests were used to detect distributed differences of categorical variables across groups. Binary logistic regression analyses were applied to explore the factors associated with awareness, knowledge, and worry about mpox. Variables with a *P* < 0.20 in the uni-variable analysis were included in the multivariable analysis. Associated factors were identified with the stepwise procedure. Odds ratios (OR) and 95% confidence intervals (CI) were calculated. A two-tailed test with a *P* < 0.05 was considered to be significant. All statistical analyses were performed in SPSS 21.0 (IBM SPSS Statistics, New York, United States).

## 3. Results

### 3.1. Characteristics of all participants

In total, 1028 community residents were included in the analysis, with a mean age of 34.70 years (standard deviation: 9.87 years). The characteristics of all participants were displayed in Table 1. Among these participants, more than two-thirds were female gender (68.3%) and well-educated (69.1% for college or above), 40.3% had local household registration, and nearly four out of five were married (78.3%) and employed (79.5%). Although approximately half of the participants earned a moderate income per month (46.3% for 5,000 ~ <10,000 RMB), over 90% of them had one or more types of health insurance (91.6%). Less than one-fifth reported smoking (16.1%) and drinking habits (13.1%), respectively, while 30.8% of them had no exercise habit. Nearly one in eight participants had ever been diagnosed with chronic diseases (12.9%) and overweight/obesity (13.0%). Notably, about 69.3% of the participants had contracted with a family doctor.

**Table 1:** Characteristics of all participants (*N* = 1,028).

**Variable**	**Number**	**Frequency (%)**
**Age (year)**		
18~30	396	38.5
31~40	404	39.3
41~60	228	22.2
**Gender**		
Male	326	31.7
Female	702	68.3
**Local household registration**		
No	614	59.7
Yes	414	40.3
**Marital status**		
Single/divorced/widow	223	21.7
Married	805	78.3
**Education level**		
High school or below	318	30.9
College or above	710	69.1
**Employment status**		
No	211	20.5
Yes	817	79.5
**Monthly income level (RMB)**		
< 5,000	270	26.3
5,000~ < 10,000	476	46.3
≥10,000	282	27.4
**Health insurance**		
No	86	8.4
Yes	942	91.6
**Active smoking**		
No	863	83.9
Yes	165	16.1
**Drinking**		
No	893	86.9
Yes	135	13.1
**Frequency of physical exercise**		
None	317	30.8
Less than once a week	336	32.7
More than once a week	375	36.5
**Ever been diagnosed with chronic diseases**		
No	895	87.1
Yes	133	12.9
**Ever been diagnosed with overweight/obesity**		
No	894	87.0
Yes	134	13.0
**Contracting with a family doctor**		
No	316	30.7
Yes	712	69.3

### 3.2. Awareness, specific knowledge of mpox

**Figure 1:**
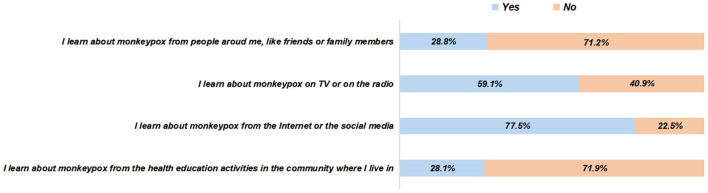
The mediums of learning about mpox among people who were aware of mpox (*N* = 801).

**Figure 2:**
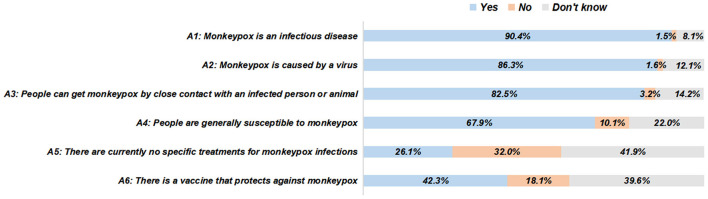
The responses to specific knowledge items of mpox among people who were aware of mpox (*N* = 801).

**Figure 3:**
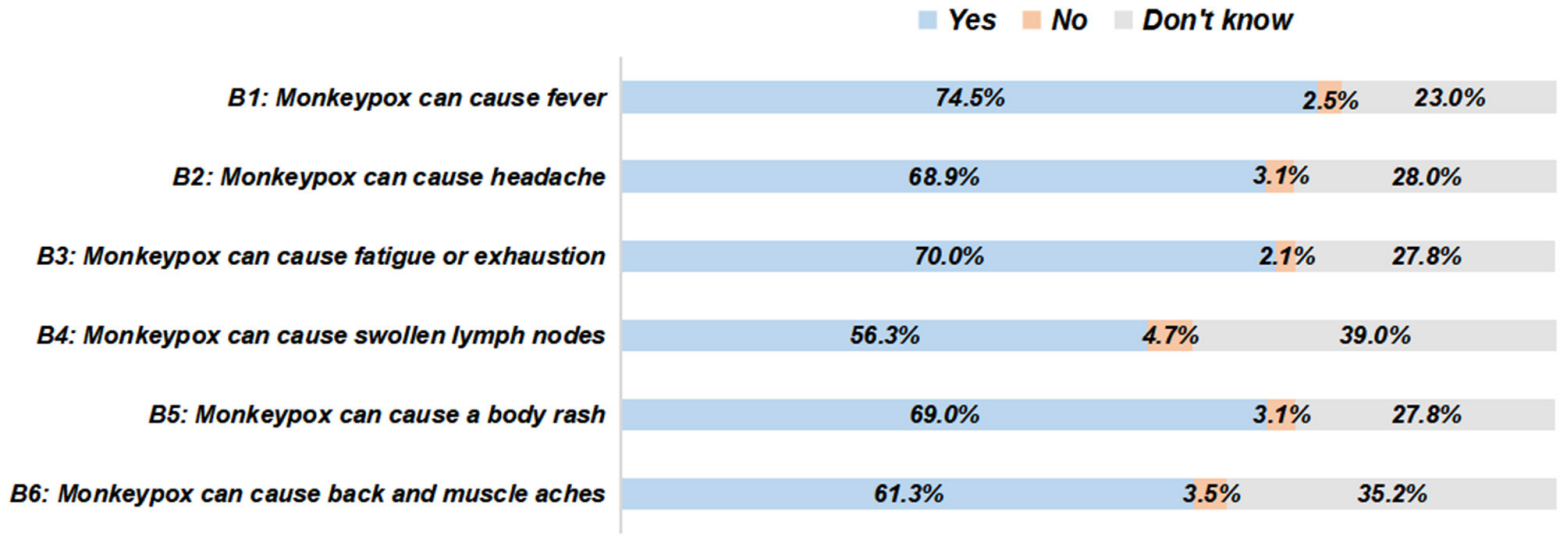
The responses to specific knowledge items of mpox-related symptoms among people who were aware of mpox (*N* = 801).

In this survey, 77.9% of the participants were aware of mpox, and 65.3% of them were aware of the global outbreak of mpox, without gender differences (Table 2). The most common medium of learning about mpox was the Internet or the social media (77.5%), followed by the TV or the radio (59.1%), people around (28.8%), and the health education activities in the community (28.1%) (Figure 1). The responses to the specific knowledge items of mpox and related symptoms demonstrated significant knowledge gaps (Figures 2, 3). Among people who were aware of mpox, the infectiousness and viral cause of mpox could be recognized by the majority (the correct rates of A1 to A3: 90.4, 86.3, and 82.5%, respectively) (Table 2). However, knowledge items regarding population susceptibility, treatments, and vaccination against mpox were not well-known (the correct rates of A4 to A6: 67.9, 26.1, and 42.3%, respectively). For mpox related symptoms, the correct rates of knowledge items ranged from 56.3 to 74.5%, in which the symptom of lymph nodes got the least awareness (Table 2).

**Table 2:** Awareness, specific knowledge, and worry about mpox in community residents (*N* = 1,028).

**Variable**	**Overall, *n* (%)**	**Male, *n* (%)**	**Female, *n* (%)**	***P*** **-values for chi-square test**
**Be aware of mpox**				
Yes	801 (77.9)	260 (79.8)	541 (77.1)	0.33
No	227 (22.1)	66 (20.2)	161 (22.9)	
**Be aware of the global outbreak of mpox**				
Yes	671 (65.3)	215 (66.0)	456 (65.0)	0.76
No	357 (34.7)	111 (34.0)	246 (35.0)	
**Specific knowledge item of mpox** ^a^				
***A1: Monkeypox is an infectious disease***				
Correct	724 (90.4)	236 (90.8)	488 (90.2)	0.80
Wrong	77 (9.6)	24 (9.2)	53 (9.8)	
***A2: Monkeypox is caused by a virus***				
Correct	691 (86.3)	222 (85.4)	469 (86.7)	0.62
Wrong	110 (13.7)	38 (14.6)	72 (13.3)	
***A3: People can get monkeypox by close contact with an infected person or animal***				
Correct	661 (82.5)	210 (80.8)	451 (83.4)	0.37
Wrong	140 (17.5)	50 (19.2)	90 (16.6)	
***A4: People are generally susceptible to monkeypox***				
Correct	544 (67.9)	179 (68.8)	365 (67.5)	0.70
Wrong	257 (32.1)	81 (31.2)	176 (32.5)	
***A5: There are currently no specific treatments for monkeypox infections***				
Correct	209 (26.1)	60 (23.1)	149 (27.5)	0.18
Wrong	592 (73.9)	200 (76.9)	392 (72.5)	
***A6: There is a vaccine that protects against monkeypox***				
Correct	339 (42.3)	123 (47.3)	216 (39.9)	**0.048**
Wrong	462 (57.7)	137 (52.7)	325 (60.1)	
**Specific knowledge item of mpox related symptoms** ^a^				
***B1: Monkeypox can cause fever***				
Correct	597 (74.5)	184 (70.8)	413 (76.3)	0.090
Wrong	204 (25.5)	76 (29.2)	128 (23.7)	
***B2: Monkeypox can cause headache***				
Correct	552 (68.9)	176 (67.7)	376 (69.5)	0.61
Wrong	249 (31.1)	84 (32.3)	165 (30.5)	
***B3: Monkeypox can cause fatigue or exhaustion***				
Correct	561 (70.0)	181 (69.6)	380 (70.2)	0.86
Wrong	240 (30.0)	79 (30.4)	161 (29.8)	
***B4: Monkeypox can cause swollen lymph nodes***				
Correct	451 (56.3)	141 (54.2)	310 (57.3)	0.41
Wrong	350 (43.7)	119 (45.8)	231 (42.7)	
***B5: Monkeypox can cause a body rash***				
Correct	553 (69.0)	174 (66.9)	379 (70.1)	0.37
Wrong	248 (31.0)	86 (33.1)	162 (29.9)	
***B6: Monkeypox can cause back and muscle aches***				
Correct	491 (61.3)	151 (58.1)	340 (62.8)	0.19
Wrong	310 (38.7)	109 (41.9)	210 (37.2)	
**Worry about mpox**				
Low	647 (62.9)	227 (69.6)	420 (59.8)	**0.002**
High	381 (37.1)	99 (30.4)	282 (40.2)	

a Only people who were aware of mpox needed to answer specific knowledge items. The answer “don't know” was considered an incorrect answer. As WHO had not yet changed the name of “monkeypox” to “mpox” during the survey time of this study, the term of “monkeypox” was applied in the survey questions.

Bold values indicated statistical significance (P < 0.05).

### 3.3. Factors associated with specific knowledge of mpox and related symptoms

Overall, 56.5 and 49.7% of the participants had a high level of knowledge regarding mpox and related symptoms, respectively. Factors associated with specific knowledge of mpox and related symptoms were identified by logistic regression analyses (Table 3). A high knowledge level of mpox was associated with being well-educated (OR: 2.03, 95%CI: 1.51~2.73), earning a high income (OR: 1.64, 95%CI: 1.12~2.41), having health insurance (OR: 1.65, 95%CI: 1.03~2.65), and doing physical exercise more than once a week (OR: 1.62, 95%CI: 1.18~2.21). A high knowledge level of mpox related symptoms was associated with having a local household registration (OR: 1.58, 95%CI: 1.22~2.05), having health insurance (OR: 1.95, 95%CI: 1.20~3.16), doing physical exercise more than once a week (OR: 1.57, 95%CI: 1.15~2.13), and contracting with a family doctor (OR: 1.39, 95%CI: 1.06~1.84). Moreover, we found that having a diagnosis history of chronic diseases was negatively associated with a high knowledge level of mpox related symptoms (OR: 0.55, 95%CI: 0.37~0.80).

**Table 3:** Factors associated with specific knowledge of mpox and related symptoms in community residents (*N* = 1,028).

**Variable**	**Knowledge level of mpox** ^a^		**Uni-variable OR (95%CI)^b^**	**Multivariable OR (95%CI)**	**Knowledge level of mpox related symptoms** ^a^		**Uni-variable OR (95%CI)^b^**	**Multivariable OR (95%CI)**
	**Low**, ***n*** **(%)**	**High**, ***n*** **(%)**			**Low**, ***n*** **(%)**	**High**, ***n*** **(%)**		
**Age (year)**								
18~30	174 (38.9)	222 (38.2)	1.00 (reference)	/	200 (38.7)	196 (38.4)	1.00 (reference)	/
31~40	161 (36.0)	243 (41.8)	1.18 (0.89, 1.57)		195 (37.7)	209 (40.9)	1.09 (0.83, 1.44)	
41~60	112 (25.1)	116 (20.0)	0.81 (0.59, 1.13)		122 (23.6)	106 (20.7)	0.89 (0.64, 1.23)	
**Gender**								
Male	138 (30.9)	188 (32.4)	1.00 (reference)	/	164 (31.7)	162 (31.7)	1.00 (reference)	/
Female	309 (69.1)	393 (67.9)	0.93 (0.72, 1.22)		353 (68.3)	349 (68.3)	1.00 (0.77, 1.30)	
**Local household registration**								
No	299 (66.9)	315 (54.2)	1.00 (reference)	/	344 (66.5)	270 (52.8)	1.00 (reference)	1.00 (reference)
Yes	148 (33.1)	266 (45.8)	**1.71 (1.32, 2.20)**		173 (33.5)	241 (47.2)	**1.78 (1.38, 2.28)**	**1.58 (1.22, 2.05)**
**Marital status**								
Single/divorced/widow	91 (20.4)	132 (22.7)	1.00 (reference)	/	106 (20.5)	117 (22.9)	1.00 (reference)	/
Married	356 (79.6)	449 (77.3)	0.87 (0.64, 1.18)		411 (79.5)	394 (77.1)	0.87 (0.65, 1.17)	
**Education level**								
High school or below	185 (41.4)	133 (22.9)	1.00 (reference)	1.00 (reference)	189 (36.6)	129 (25.2)	1.00 (reference)	/
College or above	262 (58.6)	448 (77.1)	**2.38 (1.82, 3.12)**	**2.03 (1.51, 2.73)**	328 (63.4)	382 (74.8)	**1.71 (1.31, 2.23)**	
**Employment status**								
No	109 (24.4)	102 (17.6)	1.00 (reference)	/	125 (24.2)	86 (16.8)	1.00 (reference)	/
Yes	338 (75.6)	479 (82.4)	**1.51 (1.12, 2.05)**		392 (75.8)	425 (83.2)	**1.58 (1.16, 2.14)**	
**Monthly income level (RMB)**								
< 5,000	146 (32.7)	124 (21.3)	1.00 (reference)	1.00 (reference)	158 (30.6)	112 (21.9)	1.00 (reference)	/
5,000~ < 10,000	211 (47.2)	265 (45.6)	**1.48 (1.10, 2.00)**	1.14 (0.83, 1.57)	237 (45.8)	239 (46.8)	**1.42 (1.05, 1.92)**	
≥10,000	90 (20.1)	192 (33.0)	**2.51 (1.78, 3.55)**	**1.64 (1.12, 2.41)**	122 (23.6)	160 (31.3)	**1.85 (1.32, 2.59)**	
**Health insurance**								
No	52 (11.6)	34 (5.9)	1.00 (reference)	1.00 (reference)	59 (11.4)	27 (5.3)	1.00 (reference)	1.00 (reference)
Yes	395 (88.4)	547 (94.1)	**2.12 (1.35, 3.33)**	**1.65 (1.03, 2.65)**	458 (88.6)	484 (94.7)	**2.31 (1.44, 3.71)**	**1.95 (1.20, 3.16)**
**Active smoking**								
No	371 (83.0)	492 (84.7)	1.00 (reference)	/	435 (84.1)	428 (83.8)	1.00 (reference)	/
Yes	76 (17.0)	89 (15.3)	0.88 (0.63, 1.23)		82 (15.9)	83 (16.2)	1.03 (0.74, 1.44)	
**Drinking**								
No	386 (86.4)	507 (87.3)	1.00 (reference)	/	444 (85.9)	449 (87.9)	1.00 (reference)	/
Yes	61 (13.6)	74 (12.7)	0.92 (0.64, 1.33)		73 (14.1)	62 (12.1)	0.84 (0.58, 1.21)	
**Frequency of physical exercise**								
None	159 (35.6)	158 (27.2)	1.00 (reference)	1.00 (reference)	178 (34.4)	139 (27.2)	1.00 (reference)	1.00 (reference)
Less than once a week	145 (32.4)	191 (32.9)	1.33 (0.97, 1.80)	1.23 (0.90, 1.69)	167 (32.3)	169 (33.1)	1.30 (0.95, 1.76)	1.28 (0.94, 1.76)
More than once a week	143 (32.0)	232 (39.9)	**1.63 (1.21, 2.21)**	**1.62 (1.18, 2.21)**	172 (33.3)	203 (39.7)	**1.51 (1.12, 2.04)**	**1.57 (1.15, 2.13)**
**Ever been diagnosed with chronic diseases**								
No	377 (84.3)	518 (89.2)	1.00 (reference)	/	435 (84.1)	460 (90.0)	1.00 (reference)	1.00 (reference)
Yes	70 (15.7)	63 (10.8)	**0.66 (0.46, 0.94)**		82 (15.9)	51 (10.0)	**0.59 (0.41, 0.85)**	**0.55 (0.37, 0.80)**
**Ever been diagnosed with overweight/obesity**								
No	385 (86.1)	509 (87.6)	1.00 (reference)	/	452 (87.4)	442 (86.5)	1.00 (reference)	/
Yes	62 (13.9)	72 (12.4)	0.88 (0.61, 1.27)		65 (12.6)	69 (13.5)	1.09 (0.76, 1.56)	
**Contracting with a family doctor**								
No	143 (32.0)	173 (29.8)	1.00 (reference)	/	177 (34.2)	139 (27.2)	1.00 (reference)	1.00 (reference)
Yes	304 (68.0)	408 (70.2)	1.11 (0.85, 1.45)		340 (65.8)	372 (72.8)	**1.39 (1.07, 1.82)**	**1.39 (1.06, 1.84)**

a People who were not aware of mpox were regarded with a low knowledge level.

b Variables with a *P* < 0.20 in the uni-variable analysis were included in the multivariable analysis.

Bold values indicated statistical significance (*P* < 0.05).

### 3.4. Worry about mpox and associated factors

Of the participants, 37.1% expressed a high level of worry about mpox, where females had a higher proportion than males (40.2 vs. 30.4%, *P* < 0.05) (Table 2). Factors associated with worry about mpox were also detected (Table 4). Factors that were positively associated with a high level of worry included being female (OR: 1.60, 95%CI: 1.20~2.15), having a diagnosis history of overweight/obesity (OR: 1.82, 95%CI: 1.23~2.68), and having high knowledge levels of mpox and related symptoms (OR: 1.79, 95%CI: 1.22~2.63 for a single high knowledge level; OR: 1.98, 95%CI: 1.47~2.66 for both high knowledge levels). Factors that were negatively associated with a high level of worry included having an older age (OR: 0.56, 95%CI: 0.38~0.82) and being well-educated (OR: 0.59, 95%CI: 0.44~0.80).

**Table 4:** Factors associated with worry about mpox in community residents (*N* = 1,028).

**Variable**	**Worry about mpox**		**Uni-variable** **OR (95%CI)^b^**	**Multivariable** **OR (95%CI)**
	**Low**, ***n*** **(%)**	**High**, ***n*** **(%)**		
**Age (year)**				
18~30	240 (37.1)	156 (40.9)	1.00 (reference)	1.00 (reference)
31~40	249 (38.5)	155 (40.7)	0.96 (0.72, 1.27)	0.93 (0.70, 1.25)
41~60	158 (24.4)	70 (18.4)	**0.68 (0.48, 0.94)**	**0.56 (0.38, 0.82)**
**Gender**				
Male	227 (35.1)	99 (26.0)	1.00 (reference)	1.00 (reference)
Female	420 (64.9)	282 (74.0)	**1.54 (1.16, 2.04)**	**1.60 (1.20, 2.15)**
**Local household registration**				
No	379 (58.6)	235 (61.7)	1.00 (reference)	/
Yes	268 (41.4)	146 (38.3)	0.88 (0.68, 1.14)	
**Marital status**				
Single/divorced/widow	150 (23.2)	73 (19.2)	1.00 (reference)	/
Married	497 (76.8)	308 (80.8)	1.27 (0.93, 1.74)	
**Education level**				
High school or below	189 (29.2)	129 (33.9)	1.00 (reference)	1.00 (reference)
College or above	458 (70.8)	252 (66.1)	0.81 (0.61, 1.06)	**0.59 (0.44, 0.80)**
**Employment status**				
No	129 (19.9)	82 (21.5)	1.00 (reference)	/
Yes	518 (80.1)	299 (78.5)	0.91 (0.67, 1.24)	
**Monthly income level (RMB)**				
< 5,000	156 (24.1)	114 (29.9)	1.00 (reference)	/
5,000~ < 10,000	308 (47.6)	168 (44.1)	0.75 (0.55, 1.01)	
≥10,000	183 (28.3)	99 (26.0)	0.74 (0.53, 1.04)	
**Health insurance**				
No	60 (9.3)	26 (6.8)	1.00 (reference)	/
Yes	587 (90.7)	355 (93.2)	1.40 (0.87, 2.25)	
**Active smoking**				
No	535 (82.7)	328 (86.1)	1.00 (reference)	/
Yes	112 (17.3)	53 (13.9)	0.77 (0.54, 1.10)	
**Drinking**				
No	556 (85.9)	337 (88.5)	1.00 (reference)	/
Yes	91 (14.1)	44 (11.5)	0.80 (0.54, 1.17)	
**Frequency of physical exercise**				
None	193 (29.8)	124 (32.5)	1.00 (reference)	/
Less than once a week	210 (32.5)	126 (33.1)	0.93 (0.68, 1.28)	
More than once a week	244 (37.7)	131 (37.7)	0.84 (0.61, 1.14)	
**Ever been diagnosed with chronic diseases**				
No	554 (85.6)	341 (89.5)	1.00 (reference)	/
Yes	93 (14.4)	40 (10.5)	0.70 (0.47, 1.04)	
**Ever been diagnosed with overweight/obesity**				
No	574 (88.7)	320 (84.0)	1.00 (reference)	1.00 (reference)
Yes	73 (11.3)	61 (16.0)	**1.50 (1.04, 2.16)**	**1.82 (1.23, 2.68)**
**Contracting with a family doctor**				
No	210 (32.5)	106 (27.8)	1.00 (reference)	/
Yes	437 (67.5)	275 (72.2)	1.25 (0.94, 1.65)	
**Knowledge level of mpox and related symptoms** ^a^				
Low/low	279 (43.1)	115 (30.2)	1.00 (reference)	1.00 (reference)
Low/high or High/low	105 (16.2)	71 (18.6)	**1.64 (1.13, 2.38)**	**1.79 (1.22, 2.63)**
High/high	263 (40.6)	195 (51.2)	**1.80 (1.35, 2.39)**	**1.98 (1.47, 2.66)**

a People who were not aware of mpox were regarded with a low knowledge level.

b Variables with a *P* < 0.20 in the uni-variable analysis were included in the multivariable analysis.

Bold values indicated statistical significance (*P* < 0.05).

## 4. Discussion

The multi-country outbreak of mpox in 2022 has reached the highest level of global public health alert, calling for coordination, cooperation, and global solidarity by the WHO. According to previous findings in other countries, the perception of mpox in the general population was unsatisfactory (15, 21). Although there were no mpox cases reported in China before the present survey, understanding the public awareness, knowledge, and worry about mpox helps the government and experts to develop appropriate coping strategies. This study detected the gaps in awareness and specific knowledge of mpox among community residents and the positive associations between high knowledge levels and high worry about mpox. These findings shed light on the need for community-based health education and targeted intervention.

This study identified nearly 80% of the survey participants who had ever heard of mpox and about two-thirds knew about the global outbreak. These findings showed an acceptable awareness among the general population in China, as we noticed that <50% of 651 health school students were aware of the global outbreak in Jordan (12). Only a few previous studies assessed the knowledge of mpox and most of them were targeting physicians and medical students. Researchers in Indonesia found around 10.0% of 432 general practitioners knew over 80% of the knowledge items of mpox (21 items) (13, 27). The above studies conducted in Jordan also detected unsatisfactory knowledge levels of mpox (11 items) (12). A survey among 163 Italian physicians found that only 49.7% had a general knowledge score of mpox exceeding the median (24 items) (14). A study among 558 university students found about 80% had moderate to good knowledge of mpox (knowing 60–100% of 21 items) (15). In our study, around half of the survey participants had high knowledge levels of mpox and related symptoms (correct answers ≥ 4 out of 6 items). Although a direct comparison of knowledge levels across studies cannot be conducted due to the differences in knowledge items and study populations, it's possible to identify the common gaps in mpox related knowledge. The majority of people were lack of knowledge about treatments and vaccines for mpox. In our study, only 26.1% and 42.3% of people who were aware of mpox knew no specific treatments and the existence of a vaccine against mpox, respectively. Among medical students in Jordan, only 26.2% were aware of the presence of mpox vaccination (12). This phenomenon may partly reflect public misconceptions due to the media news on the development of new drugs and vaccines that are not yet widely used. In line with previous findings, people usually learned about mpox from the Internet or social media (15). Thus, people may be easily misled by the wrong knowledge or information spread on the Internet. Public health education, especially offline education around people, should be provided along with correct, clear, and understandable messages about mpox.

In the current study, factors associated with high knowledge levels of mpox and related symptoms indicated a relatively high socioeconomic status, such as being well-educated, earning a higher income, and having health insurance. These findings were in line with those reported to be associated with knowledge of COVID-19 (28, 29). We did not find the effect of age and gender on the knowledge level, which was consistent with the findings in Italian adults (21). Whereas researchers found a positive association of age and female gender with good knowledge of mpox in a small sample of university students (15). More population-based investigations are needed to replicate these findings. We also found that people with local household registration tended to have a high level of knowledge, which may result from the better socioeconomic status of local residents than migrant residents. This result was similar to what we found in the investigation of knowledge of HPV and its vaccine (30). There were opposite effects of physical exercise and a diagnosis history of chronic diseases on specific knowledge levels in our study. The reason may partly lie in the varied attention to novel health information among physically active and inactive persons to some extent. Moreover, contracting with a family doctor seemed to help people maintain a high knowledge level. Given the facilitating effect of family doctor contracting services on health management (31), contracted people are more likely to capture up-to-date health information. Nevertheless, both active and passive access to health knowledge is of great importance for the general population.

There were limited studies that reported worry caused by mpox. A cross-sectional study among 1546 participants in Saudi Arabia detected that more than 60% of them were more worried about COVID-19 than mpox (32). In our study, a single question item was applied to measure worry about mpox and the average score was 2.96 ± 1.21. This score was relatively higher than that in Italian adults (21). The proportion of a high level of worry (very or extremely worried) was 37.1% in our study. When compared to studies similarly using a single assessment item of COVID-19 worry, the rate of high worry level tended to be lower than those among Chinese people (Guangdong: 42.05%; Henan: 58.61%) (18, 25), but higher than that among UK adults (19.8%) (26). These inconsistencies may be explained by the differences in epidemic status and the effect of prevention and control measures across countries. It's also reasonable that the influence of the mpox outbreak on the public is weaker than COVID-19 as it has not reached pandemic status. Besides, there were other kinds of tools to measure the level of worry. Some studies generally assessed the frequency and severity of worry regarding disease pandemics (16, 33), whereas other studies concentrated on assessing various worry contents, such as worries in social, economic, and life impacts, infection and related outcomes, and so on (34–36). Further studies can draw on the experience of these measurement tools on COVID-19 to explore mpox related worry.

Demographic factors associated with worry about mpox in the current study were in accordance with those found to be associated with COVID-19 worry, including age, gender, and education level (25, 37). The public worry about mpox may be partly attributed to the age and gender differences in dealing with multiple stresses and maintaining mental health (38, 39). Moreover, the influence of education level on worry about mpox may lie in that people being well-educated are more easily to obtain resources of psychological healthcare to alleviate worry. Our study also detected that people who had been diagnosed with overweight/obesity had a higher level of worry. Even though there is no evidence regarding the association between being overweight/obesity and mpox, people plagued by overweight/obesity may face more difficulties if the global outbreak becomes serious, such as increased infection risk (40, 41). It is important to note a positive association between a high level of knowledge and worry in this study. This phenomenon was also reported in other diseases (42–44). The reason for this positive association may be that people with a high knowledge level are more likely to understand the susceptibility and severity associated with mpox and the global outbreak, especially for those affected by the COVID-19 pandemic substantially.

This was a timely study to investigate public awareness, knowledge, and worry about mpox in the Chinese population. The strengths of this study included a proper sample size, a validated questionnaire, and an easily accessible survey platform with smartphones. Notably, this preliminary study may provide important prospects for community health education on mpox. The study findings help to detect vulnerable groups with poor knowledge of mpox and to establish targeted health promotion programs. Moreover, the worrying state along with the increased awareness of mpox also reminds all community health professionals to focus on psychological issues related to health education. Previous study findings suggested that suboptimal level of mpox-related knowledge could be accompanied by a high proportion of vaccine hesitancy among healthcare workers (45). It's urgently needed to conduct further investigations on the impact of public awareness, knowledge, and worry on attitudes toward mpox vaccination based on the present study sample of the general population.

This study has some limitations. First of all, the convenience sampling process may restrict the generalization of our findings to the overall general population. Moreover, the identified associations cannot help to infer causal relationships because of the cross-sectional design. Second, the assessment of mpox related knowledge was self-draft rather than a standard tool regardless of adaptation from authoritative information. Some newly identified manifestations, for example, oral mucosal enanthema (46), were not included in assessing the knowledge of mpox related symptoms. This attempt may affect the accuracy and completeness of data collection. The subjective nature of questions related to mpox may lead to over- or under-estimation of real impacts. Population-based surveys with a large sample are needed to explore comprehensive mpox knowledge scales in an objective manner. Third, the single-question measurement of worry only reflects one's perceived worry subjectively, which is not a validated diagnostic tool for measuring worry components comprehensively. It has been proposed that a single question may lower the possibility of conflating disease related worry and other existing worries (37). However, bias raised by self-reported answers could not be avoided due to the online survey design. History of psychological diseases was not collected in this study, which may also affect the worrying state of community residents to some extent. Multi-item measure scales of worry and psychological measurement are recommended to be applied in further studies. Furthermore, given that the recent mpox cases were predominantly reported in MSM, it's possible that sexual orientation may affect the perception and worry about mpox. Specific surveys targeting MSM should be conducted in the future.

## 5. Conclusions

Our study revealed room for improvement in awareness and specific knowledge of mpox in the general population in Shenzhen, China. Most people in China have no history of smallpox vaccination, and are generally susceptible to mpox virus as a result of lacking immunoprotection against infection with orthopox viruses. Health education programs should be established immediately to promote the application of knowledge-attitude-practice (KAP) in fighting against mpox at the community level. This study also highlighted a positive association between a high knowledge level and high worry about mpox. Although worry at a moderate level may help to facilitate preventive practices of mpox, timely psychological interventions are also needed to release public worry if necessary.

## Data availability statement

The raw data supporting the conclusions of this article will be made available by the authors, without undue reservation.

## Ethics statement

The studies involving human participants were reviewed and approved by the Institutional Review Board of Baoan Central Hospital of Shenzhen. The patients/participants provided their written informed consent to participate in this study.

## Author contributions

WL, JF, FR, and YX contributed to the design and implementation of the study. FR, JL, and JM collected the data. FR, RZ, WL, and JF performed the analysis and interpretation of data. FR, JL, WL, and JF drafted and revised the manuscript. All authors discussed the results and commented on the manuscript, reviewed, and approved the final manuscript to be published.
